# Multi-Axis Niche Examination of Ecological Specialization: Responses to Heat, Desiccation and Starvation Stress in Two Species of Pit-Building Antlions

**DOI:** 10.1371/journal.pone.0050884

**Published:** 2012-11-28

**Authors:** Ron Rotkopf, Erez David Barkae, Einav Bar-Hanin, Yehonatan Alcalay, Ofer Ovadia

**Affiliations:** Department of Life Sciences, Ben-Gurion University of the Negev, Be'er-Sheva, Israel; CNRS, Université de Bourgogne, France

## Abstract

Classical ecological studies discussing specialization usually focus on species’ performance along one niche axis. This approach may overlook niche differentiation evident in another dimension which could explain species co-occurrence. The present research exemplifies a comprehensive approach to examining local adaptation. Specifically, we examined multiple niche axes by subjecting a model organism to various experimental conditions to monitor responses to extreme stress associated with heat, desiccation and starvation. Our model system comprised two pit-building antlions: the habitat generalist *Myrmeleon hyalinus* and the habitat specialist *Cueta lineosa*. Previous research has shown that the foraging performance of the generalist is better than that of the specialist, even in the latter’s characteristic habitat. We illustrate that this apparent superiority of the habitat generalist does not manifest itself along other niche axes; rather, the habitat specialist holds a set of traits that provide an advantage under harsh environmental conditions. Specifically, *C. lineosa* has an advantage over *M. hyalinus* at high temperatures, exhibiting a higher survival rate and improved foraging success (i.e., high-temperature specialist). *C. lineosa* is also more efficient in its energy budget, losing less mass during starvation and gaining mass more efficiently during feeding. This superior efficiency is a result of physiological adaptations as well as behavioural responses to harsh conditions. In conclusion, our results imply that the habitat specialization of *C. lineosa* has not led it towards an evolutionary dead-end.

## Introduction

Adaptation to local biotic (e.g., competitors and predators) and abiotic (e.g., temperature, rainfall, relative humidity, etc.) conditions is important for the survival and reproduction of any organism [1]. Since different organisms have a limited set of environmental conditions under which they can better persist, local adaptations often result in specialization along one or several niche axes [2]. The degree of specialization on each axis may differ between species [3]. For instance, one species may be tolerant and reproduce under a wide range of temperatures (generalist), while another will be able to reproduce only within a limited range of temperatures (specialist) [4]. Classical studies discussing niche breadth differences between species, usually examine only one niche axis [3]. Obviously, a certain species may be a specialist on one axis (e.g., exploiting and performing well only within a narrow range of habitat types), but a generalist on another (e.g., consuming different food types with equal success, see [5]). Therefore, comparative studies should quantify inter-species variation in niche breadth by examining several different niche axes, both by observation in the field, and experimentally, by exposing the subject organisms to differing levels of environmental stress (e.g., [6]).

Temperature is one of the most important physical factors that define a species’ fundamental niche. Temperature limits some species’ distributions [7]–[9] because both extreme heat and cold can adversely affect metabolic and life-history traits [10]–[13]. Such effects should lead to optimization of metabolic and demographic traits over the range of temperatures most often experienced by a population (e.g., [8]). If this range is narrow, then thermal specialization may evolve [14]. This hypothesis frequently presumes an evolutionary trade-off between capacity to tolerate a broad range of temperatures and peak performance over a narrow range, but this trade-off lacks empirical support [15]. Selection experiments on the bacterium *Escherichia coli* have demonstrated that experimental populations always became better adapted to the temperatures at which they evolved [16]–[19]. However, their correlated responses at other temperatures varied depending on the selective temperature and were not always consistent among replicate populations maintained at the same selective temperature. For example, populations that evolved at 20°C systematically became inferior competitors at temperatures above 40°C [18], whereas five of the six populations that adapted to 42°C did not become inferior competitors at low temperatures [16]. These evolutionary experiments therefore provide ambiguous support for the existence of trade-offs in performance across the thermal niche.

High temperatures may be associated with either low or high relative humidity. These differences may be apparent when contrasting different climatic regions, such as desert vs. tropical climates, or even at smaller scales, when contrasting adjacent microhabitats [20]. In arid habitats, temperature and relative humidity are dramatically affected by shade [21]. Areas exposed to direct sunlight are characterized by both high temperature and low relative humidity, while shaded areas, albeit in desert climates, experience lower temperatures and higher relative humidity [22]. The temperature tolerance of an organism, through behavioral or physiological responses, is expected to be a crucial factor in its habitat and microhabitat choices. For instance, soil and underground environments can provide refuge from the temperature extremes which occur at the surface, while vegetation may ensure that terrestrial habitats are varied in biotic and abiotic conditions, especially for small animals like insects [21].

Water availability and temperature are the two most important abiotic variables influencing the distribution and abundance of insects [23]. Much water balance work has been undertaken in desert regions [24], demonstrating superior desiccation resistance in species from arid environments. Insects lose water by respiration, excretion and evaporation through their cuticle [23]. Desiccation resistance is generally accomplished in three ways: increasing body water content, reducing rates of water loss, or tolerating the loss of a greater proportion of body water (desiccation or dehydration tolerance) [25]. The standard method of measuring desiccation resistance is to record the mass change of insects maintained in dry conditions [23]. Losses represent water and also dry matter metabolized as CO_2_. Insects maintained in dry air experience starvation and desiccation, whereas those maintained at higher humidity levels experience mainly starvation.

Small body size is commonly accepted as disadvantageous for insects in terrestrial environments because of their small water storage capacity, combined with a relatively large surface area, that enables faster cutaneous water loss [20]. However, small body size can enable the organism to experience finer spatial heterogeneity among different microclimatic conditions within the same general habitat, taking advantage of the August average daily temperature: 25.7°C, average daily maximum: 32.8°C, microclimates [20]. Such an ability to switch microclimates may be restricted in insects characterized by limited movement, such as sit-and-wait and trap-building predators, forcing them to adapt more specifically to their local environment [26]. For example, pit-building antlions are dramatically affected by their physical environment, showing differences in pit size, body size, growth trajectory and life-history plasticity, depending on the local conditions in their habitat of origin [27], [28]. Additionally, antlion activity is, to a large extent, constrained by high temperatures [29].

Different species of antlions show major differences in their microhabitat preferences – soil type, particle size, shade, etc. [30]–[34]. Lucas [22] compared two species of antlions, *Myrmeleon crudelis* and *M. carolinus*, occupying shaded and open habitats, respectively, and found evidence for physiological adaptations to harsh environments in *M. carolinus*, at the expense of a reduced survivorship when interacting with other species. However, despite their preferences for different soil types, pit-building antlions may also construct pits in less desirable habitats [33], [35], [36]. In this study we used two species of pit-building antlions, both highly common in Israel. The habitat generalist, *Myrmeleon hyalinus* Olivier, 1811, inhabits and performs equally well in both sand- and loess-derived soils [33], [37], while the habitat specialist, *Cueta lineosa* Rambur, 1842, inhabits only fine-grained soils such as loess [37], while showing reduced foraging performance in coarse-grained sandy soils [33]. Sand-derived soils (i.e., coarse-grained texture) in the Israeli Negev desert are surrounded and fragmented by loess-derived soils (i.e., fine-grained texture), forming a wide range of habitat mosaics ([38], pp. 43–46). Furthermore, as predicted by the inverse texture hypothesis, in the semi-arid and arid regions of Israel, the former soils are much more productive than the latter ones [39]. This may explain why *M. hyalinus* larvae originating from desert habitats exhibit strong selectivity to sand [33] and why their natural abundance in sand-derived soils is higher than in loess-derived soils [37]. *M. hyalinus*’ superior performance in both soil types seemed to show only part of the picture. An additional important difference in these species’ natural occurrence is their microhabitat selection: *M. hyalinus* is usually found under trees or bushes (i.e., shaded microhabitat), while *C. lineosa* is found in open areas, exposed to direct sunlight. Since niche theory predicts that co-occurring species should show some level of niche separation [3], we predicted that *C. lineosa* should perform better than *M. hyalinus* along other environmental axes, related to arid conditions – i.e., high temperature, low relative humidity, and poor food abundance.

Low and stochastic food abundance are characteristic of desert conditions, and animals originating from such conditions are expected to handle starvation periods better than animals from more benign habitats (e.g., [40]). Animals respond to starvation or shortage of prey in two main ways: they can reduce their metabolic rate and wait for prey abundance to increase, or alternatively, they can maintain their current metabolic rate and actively search for better foraging sites [41], [42]. These two strategies are context-dependent; reducing metabolic rate is possibly preferred under harsh (extreme temperatures or low prey encounter rates) or stochastic conditions (e.g., [43]). Alternatively, increased activity may be preferred under higher prey encounter rates or more predictable conditions [26], [41], [44]. Clearly, these responses may also be related to other individual traits, such as body size or developmental stage [42]. Starvation endurance is an important trait, especially in sit-and-wait predators, which suffer from fluctuations in prey abundance much more than closely related actively searching predators [45]. Like other sit-and-wait predators, such as spiders, antlions are capable of dramatically reducing their metabolic rates [46], [47]. Since not all species and populations experience the same fluctuations in prey abundance [48], the response to starvation may be dependent on habitat-of-origin. Indeed, Arnett and Gotelli [28] have illustrated that antlion populations originating from temperate regions had better starvation endurance than populations originating from sub-tropical regions.

We hypothesized that antlion species originating from harsher environments, which experience less favorable conditions and lower prey encounter rates, should be less sensitive to starvation. Specifically, we expected *M. hyalinus*, inhabiting shaded microhabitats, to lose body mass more rapidly during starvation than *C. lineosa*, which inhabits open areas, characterized by higher temperatures, lower relative humidity, and a low and stochastic prey encounter rate. Physiological differences in the ability of individuals to cope with starvation may be attributed to genetic differences, phenotypic plasticity or a combination of both. To test the effect of these factors, individual antlions’ ability to handle starvation stress should be examined under several different habitat conditions (e.g., temperature and relative humidity). If each species’ response to starvation is affected mainly by its local conditions, then we expected both species to respond in similar fashions when exposed to the same climatic conditions. However, it should be mentioned that G×E interactions may effectively 'hide' genetic differences under certain environmental conditions [49]. Alternatively, if responses to starvation are genetically fixed or canalized, we expected to see differences in pit-building behaviour and relative growth rate between the species, even when kept under the same conditions.

## Methods

### Study Species and Habitats-of-origin

We collected *M. hyalinus* larvae under different tamarisk trees located in Nahal Secher (N 31°06′, E 34°49′), a sandy area 15 km south of the city of Be'er-Sheva, Israel, and brought them to the laboratory. *M. hyalinus* is the most abundant pit-building antlion in Israel [37]. The larvae attain maximal lengths of about 10 mm and body masses of up to 0.06 g before pupating [27]. This species performs equally well in sand-derived and loess-derived soils [33]. The fact that in our research area sandy soils are more productive than loess-derived soils [39], may explain why this species is found in higher abundance in the former coarse-grained soils [37]. In addition, we collected *C. lineosa* larvae from the loessial plains near Be’er-Sheva (N 31°16′, E 34°50′). Occurring mainly in the Israeli Negev desert, *C. lineosa* also exists in several small populations located in central and northern Israel, but is restricted to light soils, such as loess [37]. This species is a habitat specialist: its performance declines when placed in coarse-grained soils [33]. The two antlions are similarly sized and have comparable life cycles. Although they largely overlap in their geographical distribution, they rarely overlap in their microhabitat use. Specifically, *M. hyalinus* prefers shaded microhabitats [37], [50], while *C. lineosa* is mainly found in open microhabitats exposed to direct sunlight [37]. Therefore, it is unlikely that interference competition exists between the two antlion species, even in loess-derived soils. However, it is possible that they indirectly compete for their arthropod prey (i.e., exploitation competition). All required permits and approvals for this work were obtained from Israel’s Nature and National Parks Protection Authority, permit no. 2010/37830. In compliance with all the relevant laws and regulations prevailing in Israel, self-regulation and accountability of local programs by an Institutional Animal Care and Use Committee (IACUC) are not applicable for the use of invertebrates in research (Israel's Animal Welfare Act 1984).

### Experimental Procedures

The study was comprised of three complementary experiments: 1) Thermal stress experiment, investigating the behavioral responses and growth efficiency of the two species when exposed to increased temperatures, while also testing if these effects are consistent when using more realistic prey items such as ants, under field-representative temperatures. 2) Gradual heating experiment, to test whether the antlions respond differently to gradual heating vs. sudden heating. 3) Starvation and humidity experiment, testing responses to starvation under different relative humidity levels. In all the experiments, fresh antlions were collected in the field and kept separately in round plastic cups (10.5 cm diameter, 7 cm height) filled with 3 cm of sand (for *M. hyalinus*) or loess (for *C. lineosa*). Antlion larvae were fed with mealworms *ad libitum* until the beginning of the experiments (habituation period). Larvae were weighed throughout the experiments to ±0.1 mg (CP224S, Sartorius AG, Goettingen, Germany).

#### Thermal stress experiment

After the habituation period, all the antlions (*M. hyalinus* and *C. lineosa*, N = 30 per treatment per species) were starved for one week. This procedure was used to standardize the hunger level of the antlions. It ensures that the physiological state of all antlion larvae is approximately the same at the beginning of the experiment. Thereafter, we put the antlions into a controlled environmental chamber (25°C, 30% RH, day:night length 12∶12 h) for three days, after which they were each fed with a single mealworm. The antlions were then exposed to three days of high temperature (30°C, 40°C, 45°C or 50°C), and then fed another mealworm. An exception is one treatment in which the antlions were exposed to 45°C for only 24 hours. A temperature of 45°C for three days was found to be a lethal treatment for *M. hyalinus*, but not for *C. lineosa*, therefore the shorter treatment (in which both species survived) was added. At the end of the period of thermal stress, the temperature was reduced to 25°C for three days, and the larvae were fed again. By the end of each of the three phases, we monitored pit building activity, pit diameter, response to a single mealworm prey (yes/no), response time to prey (within those who responded, similarly to [51]), and growth efficiency (i.e., conversion of prey mass to predator mass). Growth efficiency was measured by dividing the difference in antlion body mass before and after feeding by the prey body mass (measured before feeding) 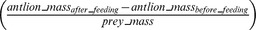 [52]. To this end, the antlions were weighed ca. three hours after feeding, to ensure that most antlions will have consumed their prey. Body mass loss during this three-hour period is negligible, as antlions do not defecate. The meal is not fully consumed, therefore growth efficiency values usually range between 0 (no consumption) and 0.9 (near-complete consumption of the mealworm).

Survival rate was compared between species for each temperature treatment using Pearson’s Chi-square. The proportion of antlions that constructed pits, and the proportion of antlions that responded to prey, was compared between periods (before, during and after the heating period) within each treatment using Pearson’s Chi-square. Pit diameter, response time to prey and growth efficiency were similarly compared between periods using Wilcoxon’s signed-rank test.

The second phase of this experiment aimed at examining differences in foraging performance when using natural ant prey, while also simulating more realistic field-representative temperatures. We thus collected fresh antlions and ants (*Pheidole pallidula* and *Messor aegyptiacus*) from the same locations described above. After the habituation period, all individuals were starved for one week. Thereafter, we placed the antlions in a controlled environmental chamber (30% RH, day:night length 12∶12 h) for three days of high temperature (30°C or 40°C), after which they were each fed with a single ant. We measured each antlion’s response time to prey, and whether it succeeded in capturing the ant prey. Response time was compared between species and temperatures using ANOVA GLM, using the residuals after regressing response times against antlion body mass. Prey capture success was compared between species and temperatures using logistic regression. While temperatures in the first phase of this experiment were selected to exert the highest stress possible and examine the lethal temperature limit, milder, more realistic field temperatures were chosen in the second phase: 30°C being close to the mean temperature near Be’er-Sheva during the summer months (August average daily temperature: 25.7°C, average daily maximum: 32.8°C, [53]), and 40°C as a representative temperature of open areas, exposed to direct radiation. Soil temperatures in areas exposed to sunlight are higher than the surrounding air temperature: average daily highs of 36.1°C in soil and 33.2°C in air were recorded in Be’er-Sheva [53], and daily highs of 43°C in soil and 33°C in air were recorded in the central Negev [54].

#### Gradual heating experiment

After the habituation period, all the antlions (*M. hyalinus* and *C. lineosa*, N = 30 per treatment per species) were starved for one week. Thereafter, all antlions were placed into one of two controlled environmental chambers (30°C, 30% RH, day:night length 12∶12 h) for seven days. In the first chamber, the temperature was kept stable for 3.5 days, after which it was abruptly increased to 40°C for the remaining 3.5 days. In the second chamber, the temperature was gradually and linearly increased (over the entire experimental period) to 40°C. We weighed the antlions at the beginning and end of the experiment and measured their pit diameter periodically. At the end of the experimental period each antlion was fed a single mealworm in order to measure response time to prey and growth efficiency. Antlions were kept in plastic cups (10.5 cm diameter) filled with 3 cm of sand (for *M. hyalinus*) or loess (for *C. lineosa*). To achieve a better understanding of the changes in body mass throughout the experiment, while minimizing pit disturbance, an additional group of antlions was kept in separate cups, under the same experimental conditions. Antlions in this second group were weighed more frequently, but their pit diameter was not measured, because of the frequent disturbance caused by the weighing. Relative growth rate was calculated according to the common formula (e.g., [55], [56]) 
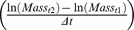
. In the disturbed cups, relative growth rate was compared between the earlier, cooler 3.5 days, and the later, warmer 3.5 days by repeated measures ANOVA. In the undisturbed cups, relative growth rate (i.e., mass loss) and pit diameter were compared between species and treatments using repeated measures ANOVA. Growth efficiency and response time were measured only at the end of the experimental period, and were thus analysed using ANOVA.

#### Starvation and humidity experiment

To impose a continuous and steady desiccation stress, we placed antlions from both species (N = 30 per treatment per species) into one of two climate chambers, both set to the same temperature of 30°C (day:night length 12∶12 h), but to different relative humidity levels –30% (9.52 mmHg) vs. 70% (22.22 mmHg). This temperature was selected as it is close to daily average temperatures during the summer months, and takes into account the larvae’s inability to escape warm temperatures by burrowing deeper into the soil. Although field temperatures may reach peaks of over 40°C, they also decrease dramatically during the night. Since we wished to impose a steady but survivable desiccation stress, we chose a temperature closer to the daily mean, rather than the daily maximum. The antlions were starved for 60 days, while control groups were fed one mealworm per week. We weighed the antlions and measured their pit diameter periodically throughout the experiment. Also, at the beginning and end of the starvation period, one pre-weighed mealworm was given to each antlion in order to measure response time to prey, and growth efficiency. Pit diameter, response time, growth efficiency and relative growth rate (or mass loss) were compared between species and treatments using ANOVA GLM.

All statistical analyses were carried out using STATISTICA, ver. 8.0.

## Results

### Thermal Stress Experiment

Survival rate did not differ significantly between species in the milder treatments: survival rates for *M. hyalinus* were 29/30 (96.7%), 30/30 (100%), and 29/30 (96.7%) for 30°C, 40°C and 45°C/24 h respectively, and for *C. lineosa* 28/30 (93.3%), 29/30 (96.7%) and 27/30 (90.0%) for 30°C, 40°C and 45°C/24 h respectively (Pearson’s Chi-square, P = N.S. for all comparisons). In the harsher treatment of 45°C/72 h, survival rates were 24/30 (80.0%) in *C.lineosa*, but no *M. hyalinus* survived (χ^2^_1_ = 40, P<0.001). In the 50°C treatment, no antlions survived from either species.

The tendency to construct pits (Table 1) was consistent for most temperatures between the pre-heat and heating period (Pearson Chi-Square, P = N.S.). Only in one case (*M. hyalinus*, 45°C/24 h) was there a significant decrease in the proportion of antlions that constructed pits (χ^2^_1_ = 11, P<0.001). When comparing pit-building proportions before and after the heat period, no significant differences were found (Pearson Chi-Square, P = N.S. for all comparisons), indicating that the heat period caused no lingering effects.

**Table 1:** Proportion of antlions that constructed pits.

Species	Temperature (°C)	Before heat (25°C)	During heat	After heat (25°C)
*C. lineosa*	30	26/30 (86.7%)	27/30 (90.0%)	23/28 (82.1%)
	40	29/30 (96.7%)	28/30 (93.3%)	27/29 (93.1%)
	45/24 h	20/27 (74.1%)	23/28 (82.1%)	22/27 (81.5%)
	45/72 h	27/30 (90.0%)	27/28 (96.4%)	24/24 (100%)
*M. hyalinus*	30	26/30 (86.7%)	28/29 (96.6%)	24/29 (82.8%)
	40	26/30 (86.7%)	27/30 (90.0%)	24/30 (80.0%)
	45/24 h	30/30 (100%)	20/29 (69.0%)	29/29 (100%)

The pit-building proportion during heat did not change for most temperatures, compared to the proportion before heat (Pearson Chi-Square, P = N.S.). Only in one case (*M. hyalinus*, 45°C/24 h) was there a significant decrease in the proportion of pit-building (χ^2^_1_ = 11,P<0.001). When comparing pit-building proportions before and after the heat period, no significant differences were found (Pearson Chi-Square, P = N.S. for all comparisons).

**Figure 1:**
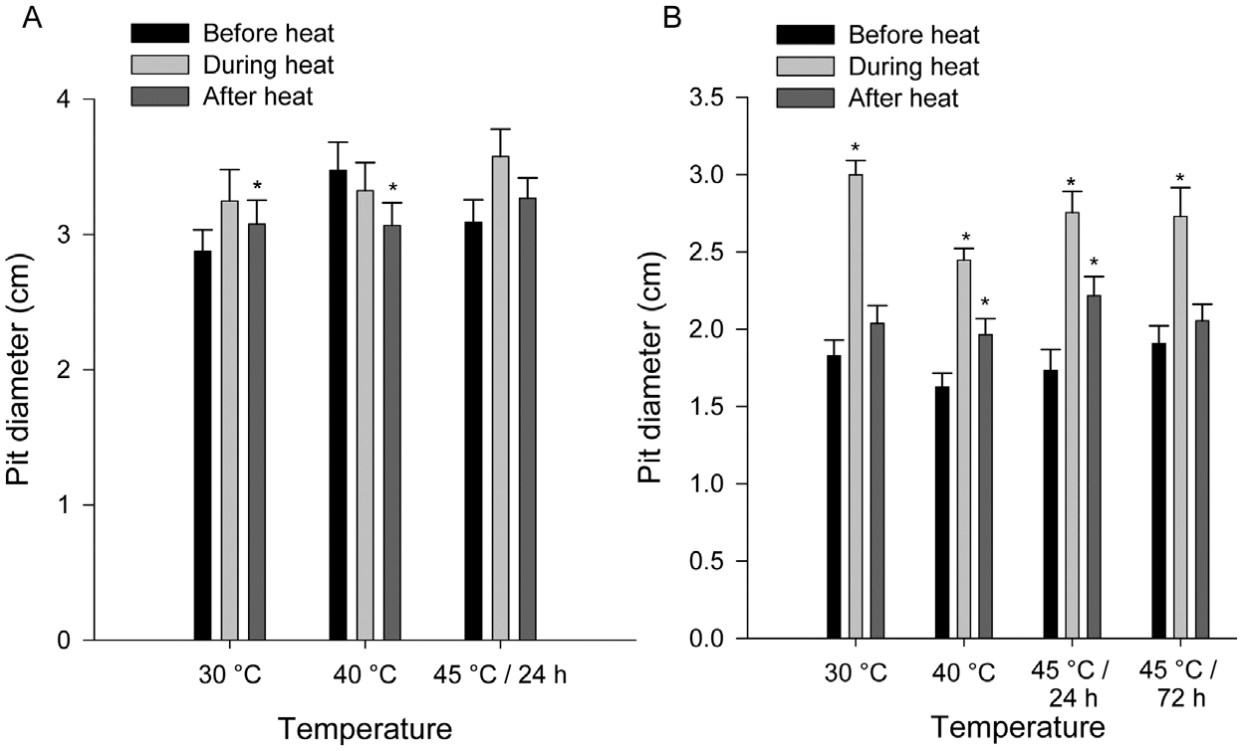
Pit diameter in (a) *M. hyalinus* and (b) *C. lineosa*. Pit diameter was measured before, during and after exposure to high temperatures. In *M. hyalinus*, pit diameter did not change during the high-temperature period. In *C. lineosa*, pit diameter increased in all temperatures, compared to the initial temperature of 25°C. Asterisks mark significant differences from the pit diameter before heat (Wilcoxon paired-ranks test, P<0.05).

In *M. hyalinus* (Fig. 1a), pit diameter did not change significantly during the high-temperature period (Wilcoxon, P = N.S. for all treatments). Pit diameter after returning to 25°C slightly increased in the 30°C treatment (P = 0.046) and slightly decreased in the 40°C treatment (P = 0.023). In *C. lineosa* (Fig. 1b), pit diameter increased during the high-temperature period in all treatments, compared to the initial temperature of 25°C (P<0.001 for all treatments). When returning to 25°C, pit sizes decreased, but were still higher than before the heat exposure in the 40°C and 45°C/24 h treatments (P = 0.025 and 0.010, respectively).

The proportion of antlions that responded to prey (Table 2) was largely consistent between the pre-heat and heating periods (Pearson Chi-Square, P = N.S.). The proportion decreased significantly in *C. lineosa* only under the most extreme treatment (45°C/72 h, χ^2^_1_ = 5.9, P = 0.015), and returned to its original level when the temperature was restored to 25°C. In *M. hyalinus*, the proportion decreased significantly in the 45°C/24 h treatment (χ^2^_1_ = 25.4, P<0.001). The proportion increased back when the temperature was restored to 25°C, but remained lower than the original level (χ^2^_1_ = 4.26, P = 0.039).

**Table 2:** Proportion of antlions that responded to prey.

Species	Temperature (°C)	Before heat (25°C)	During heat	After heat (25°C)
*C. lineosa*	30	26/30 (86.7%)	27/30 (90.0%)	23/28 (82.1%)
	40	29/30 (96.7%)	27/30 (90.0%)	27/29 (93.1%)
	45/24 h	20/28 (71.4%)	22/28 (78.6%)	21/27 (77.8%)
	45/72 h	27/30 (90.0%)	17/27 (63.0%)	23/24 (95.8%)
*M. hyalinus*	30	26/30 (86.7%)	28/29 (96.6%)	24/29 (82.8%)
	40	26/30 (86.7%)	26/30 (86.7%)	24/30 (80.0%)
	45/24 h	29/30 (96.7%)	10/29 (34.5%)	23/29 (79.3%)

The proportion of antlions that responded to prey did not change significantly for most treatments, compared to the proportion before heat (Pearson Chi-Square, P = N.S.). The proportion decreased significantly in *C. lineosa* only under the most extreme treatment (45°C/72 h, χ^2^_1_ = 5.9, P = 0.015), and returned to its original level when the temperature was restored to 25°C. In *M. hyalinus*, the proportion decreased significantly in the 45°C/24 h treatment (χ^2^_1_ = 25.4, P<0.001). The proportion increased back when the temperature was restored to 25°C, but remained lower than the original level (χ^2^_1_ = 4.26, P = 0.039).

**Figure 2:**
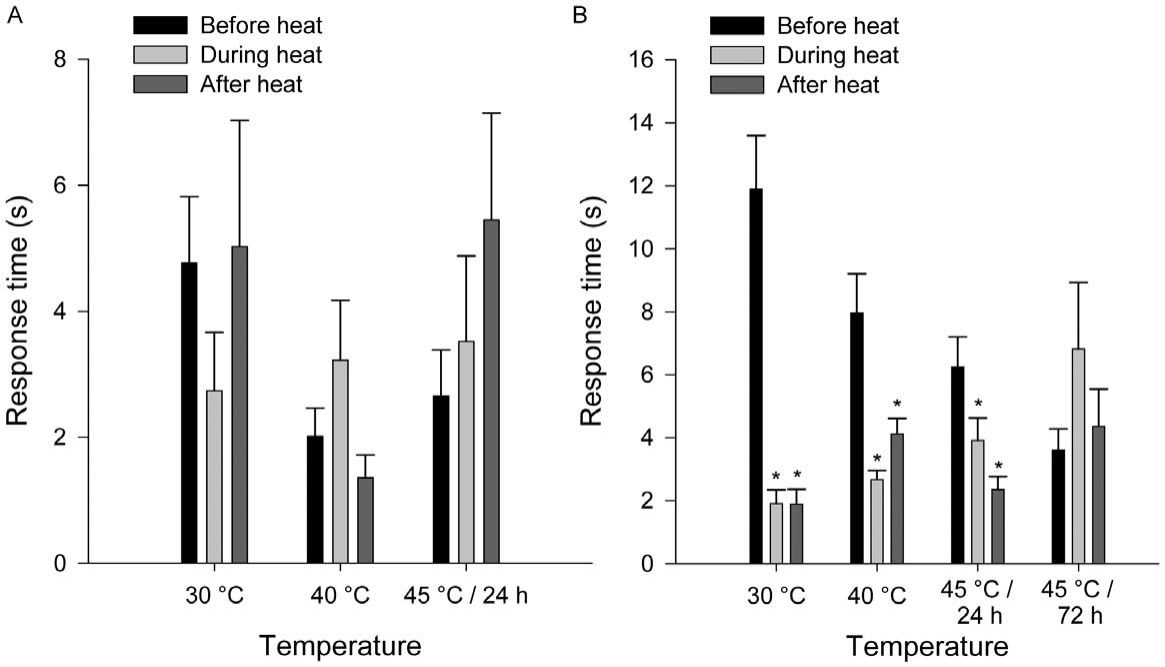
Response time to prey in (a) *M. hyalinus* and (b) *C. lineosa*. Response time was measured before, during and after exposure to high temperatures. In *M. hyalinus*, response time did not change during the high-temperature period or after returning to 25°C, compared to the initial 25°C period (Wilcoxon paired-ranks test, P = N.S. for all treatments). In *C. lineosa*, response time decreased in three out of the four treatments, (30°C: P<0.001, 40°C: P = 0.004, 45°C/24 h: P = 0.026), and remained lower than original levels in all these three treatments after returning to 25°C (30°C: P<0.001, 40°C: P = 0.011, 45°C/24 h: P = 0.007). In the most extreme treatment (45°C/72 h) no differences were found in response time between periods. Asterisks mark significant differences from the response time before heat (Wilcoxon paired-ranks test, P<0.05).

In *M. hyalinus* (Fig. 2a), response time to prey was consistent between the pre-heat, heating and post-heat periods (Wilcoxon paired-ranks test, P = N.S. for all treatments). In *C. lineosa* (Fig. 2b), response time decreased in three out of the four treatments (30°C: P<0.001, 40°C: P = 0.004, 45°C/24 h: P = 0.026), and remained lower than original levels in all these three treatments, after returning to 25°C (30°C: P<0.001, 40°C: P = 0.011, 45°C/24 h: P = 0.007). In the most extreme treatment (45°C/72 h) no differences were found in response time between periods.

In *M. hyalinus,* growth efficiency (the ability to convert prey mass to predator mass) decreased significantly during the heating period in the 30°C (from 0.50±0.06 to 0.25±0.06, Wilcoxon signed-ranks test, P = 0.009) and the 45°C/24 h (from 0.57±0.05 to 0.24±0.07, P = 0.003) treatments, but returned to original levels when the temperature was restored to 25°C. In *C. lineosa*, growth efficiency increased significantly during heat in the 30°C treatment (from 0.25±0.07 to 0.55±0.08, P = 0.03), and decreased significantly during heat in the 45°C/72 h treatment (from 0.25±0.06 to −0.11±0.10, P = 0.005). In all the treatments, growth efficiencies returned to their original levels when temperature was restored to 25°C.

**Figure 3:**
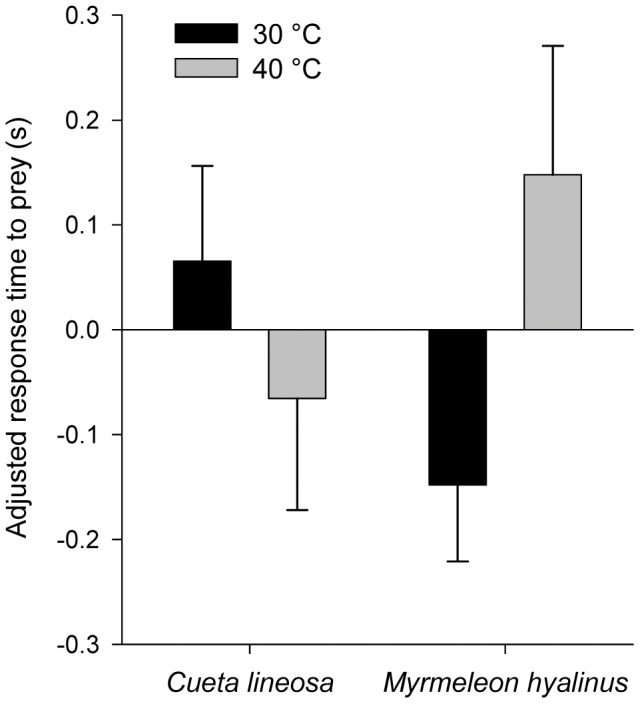
Response time (corrected values – residuals after regressing with body mass) of *C. lineosa* and *M. hyalinus*. *C. lineosa* responded faster to prey (live ants) when exposed to 40°C, while *M. hyalinus* responded faster to prey when exposed to 30°C (GLM, Temperature×Species interaction, F_1,232_ = 4.535, P = 0.034).

**Figure 4:**
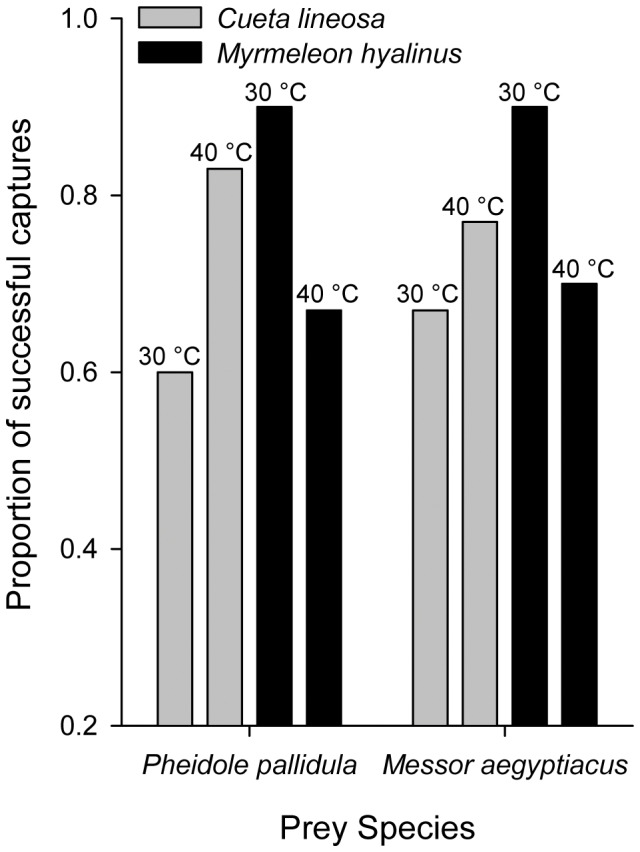
Proportion of successful ant captures in *C. lineosa* and *M. hyalinus*. *C. lineosa* were more successful in capturing prey when exposed to 40°C, while *M. hyalinus* were more successful when exposed to 30°C. (Logistic regression, p = 0.001 for the Temperature × Species interaction). No difference was found between the effects of the two ant species.

When given ants as prey items, *C. lineosa* responded faster (Fig. 3) when exposed to 40°C, while *M. hyalinus* responded faster when exposed to 30°C (F_1,232_ = 4.535, P = 0.034; Temperature × Species interaction). Similarly, *C. lineosa* were more successful in capturing prey when exposed to 40°C, while *M. hyalinus* were more successful when exposed to 30°C. (Fig. 4, Logistic regression, Temperature × Species interaction, P = 0.001). These patterns were consistent with both prey species.

### Gradual Heating Experiment

**Figure 5:**
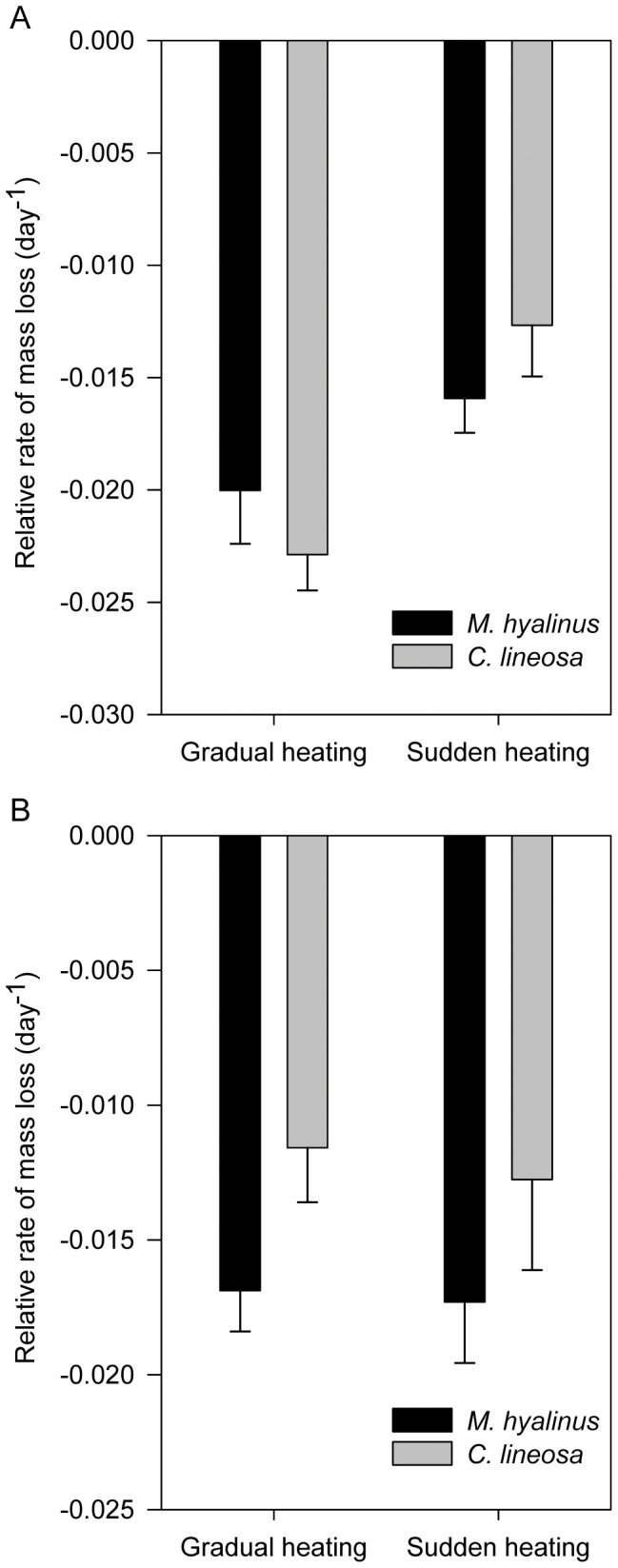
Relative rate of mass loss during heating in (a) disturbed cups and (b) undisturbed cups. Antlions kept in the disturbed cups (a) lost mass faster when exposed to gradual heating (F_1,114_ = 12.93, P<0.001). No difference was found between species (F_1,114_ = 0.01, P = 0.922). *M. hyalinus* showed a slightly higher mass loss rate in the gradual heating treatment (F_1,57_ = 2.051, p = 0.158), while in *C. lineosa* this trend was statistically significant (F_1,57_ = 13.603, p<0.001), leading to a marginally significant interaction term (F_1,114_ = 2.37, P = 0.13; Species×Treatment interaction). In the undisturbed cups (b), *M. hyalinus* lost mass faster than *C. lineosa* (F_1,113_ = 4.24, P = 0.042), and no difference was found between treatments (F_1,113_ = 0.11, P = 0.737).

**Figure 6:**
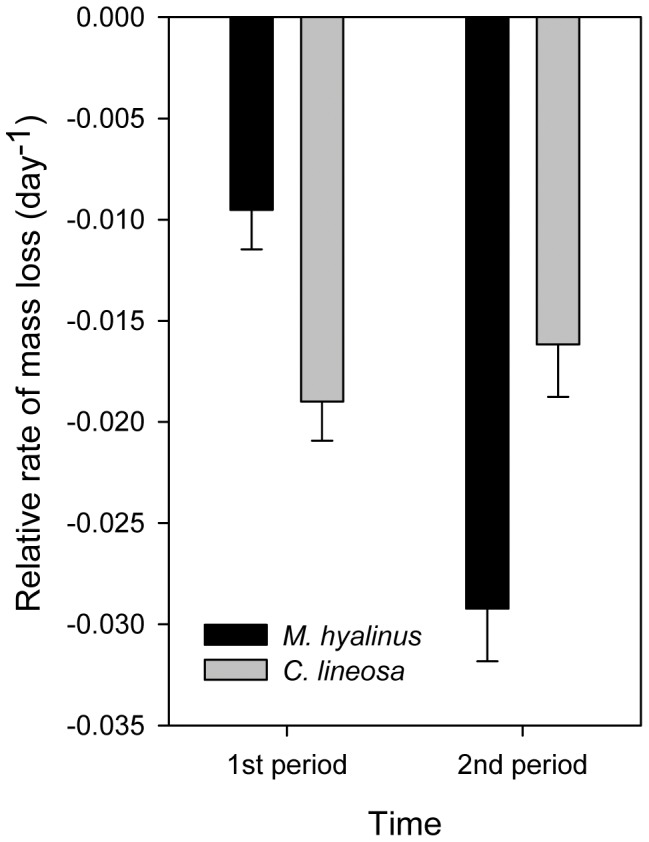
Relative rate of mass loss during heating separated into periods. *M. hyalinus* lost mass at a slower rate in the earlier, cooler period, but its rate of mass loss increased dramatically in the warmer period. *C. lineosa*’s mass loss rate remained relatively constant throughout the experiment (repeated measures, F_1,114_ = 20.21, P<0.001; Time×Species interaction).

Antlions in the group that was weighed and disturbed more frequently (Fig. 5a) lost mass faster when exposed to gradual heating (F_1,114_ = 12.93, P<0.001). No difference was found between species (F_1,114_ = 0.01, P = 0.922). *M. hyalinus* showed a slightly higher mass loss rate in the gradual heating treatment (F_1,57_ = 2.051, P = 0.158), while in *C. lineosa* this trend was statistically significant (F_1,57_ = 13.603, P<0.001), leading to a marginally significant interaction term (F_1,114_ = 2.37, P = 0.13; Species×Treatment interaction). However, when separating the mass loss rate into two equal periods: the earlier, cooler 3.5 days vs. the later, warmer 3.5 days (Fig. 6), we found that the rate of mass loss in *M. hyalinus* increases dramatically in the warmer period, while *C. lineosa* exhibits a relatively stable mass loss rate between periods (repeated measures, F_1,114_ = 20.21, P<0.001; Time×Species interaction). In the undisturbed cups (Fig. 5b), *M. hyalinus* lost mass faster than *C. lineosa* (F_1,113_ = 4.24, P = 0.042), and no difference was found between treatments (F_1,113_ = 0.11, P = 0.737). Growth efficiency of *M. hyalinus* at the end of the experiment (0.48±0.03) was significantly higher than that of *C. lineosa* (0.31±0.04, F_1,109_ = 9.53, P = 0.003). No differences were found between treatments (F_1,109_ = 1.50, P = 0.224). Concerning response time, generally, *M. hyalinus* responded to prey faster (3.14±0.56 s) than *C. lineosa* (4.62±0.79 s, F_1,65_ = 4.45, P = 0.039). Response times in the gradual heating treatment decreased in *C. lineosa* (3.42±0.67 s), and increased in *M. hyalinus* (4.13±0.87 s), compared to the sudden heating treatment (*C. lineosa*: 6.69±1.67 s; *M. hyalinus*: 2.1±0.64 s; F_1,65_ = 8.33, P = 0.005; Species×Treatment interaction). Pit diameter of *M. hyalinus* (4.33±0.19 cm) was larger than that of *C. lineosa* (2.41±0.11 cm; F_1,88_ = 55.83, P<0.001). However, the pit diameter of *M. hyalinus* decreased over the time of exposure, while the pit diameter of *C. lineosa* remained unaffected (repeated measures, F_1,88_ = 18.67, P<0.001; Time×Species interaction).

### Starvation and Humidity Experiment

In the starvation treatments, pit diameter of *M. hyalinus* (51.25±0.97 mm) was larger than that of *C. lineosa* (21.87±0.25 mm, F_1,69_ = 392.86, P<0.001). In general, pit diameter decreased over time (F _4,276_ = 7.25, P<0.001), but this trend was less prominent in the humid-air treatment (F _4,276_ = 2.16, P = 0.07; Treatment×Time interaction) and more prominent in *M. hyalinus*, as opposed to *C. lineosa*, which did not show significant changes in pit diameter over time (F _4,276_ = 8.91, P<0.001; Species×Time interaction). Pit diameter of *M. hyalinus* was larger in the humid-air treatment (56.18±1.12 vs. 46.78±1.40 mm, F_1, 29_ = 18.2409, P<0.001), while *C. lineosa* showed no significant difference between treatments (F_1, 39_ = 2.267, P = 0.140). In the control groups (which were fed regularly), high variances prevented finding significant differences in pit diameter between species (*M. hyalinus*: 86.29±23.79 mm, *C. lineosa*: 32.06±31.03 mm; F_1, 33_ = 0.532, P = 0.471) or between treatments (dry air: 70.64±28.04 mm, humid air: 47.71±27.26 mm; F_1, 33_ = 0.320, P = 0.575).

*M. hyalinus* responded to prey faster than *C. lineosa* (1.94±0.78 vs. 6.40±0.83 s; F_1,59_ = 23.62, P<0.001), and response times in the humid-air treatment were longer than in the dry-air treatment (5.69±0.81 vs. 2.65±0.79 s; F_1,39_ = 7.1, P = 0.01). Among starved *C. lineosa*, response times were shorter after the starvation (5.49±1.10 vs. 7.26±1.11 s before starvation), while in *M. hyalinus* they were slightly longer (2.36±0.95 vs. 1.09±0.29 s before starvation; F_1,19_ = 4.9065, P = 0.03; Time×Species interaction). This trend was more prominent in the humid-air treatment. In the control (fed) groups, relative humidity was not found to affect response time (F_1,20_ = 0.65, P = 0.43), but, as in the starved groups, *M. hyalinus* responded faster to prey (2.59±1.01 vs. 7.90±1.31 s in *C. lineosa*; F_1,20_ = 10.30, P<0.001).

We found no difference in growth efficiency between species (F_1,66_ = 2.605, P = 0.111) or between treatments (F_1,66_ = 0.728, P = 0.397). No such differences were found in the control group, either (Species: F_1,42_ = 1.396, P = 0.244; Treatment: F_1,42_ = 0.324, P = 0.572).

**Figure 7:**
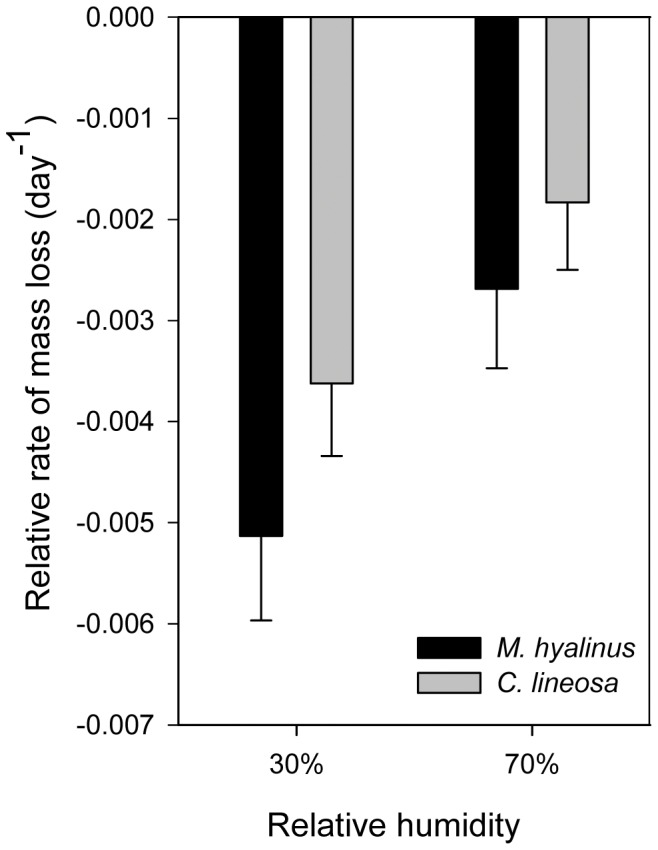
Relative rate of mass loss in starved antlions. In both species, increased relative humidity decreased the rate of body mass loss of starved individuals (F_1,67_ = 22.46, P<0.001). *C. lineosa* lost mass at a slower rate than *M. hyalinus* (F_1,67_ = 7.00, P = 0.01).

**Figure 8:**
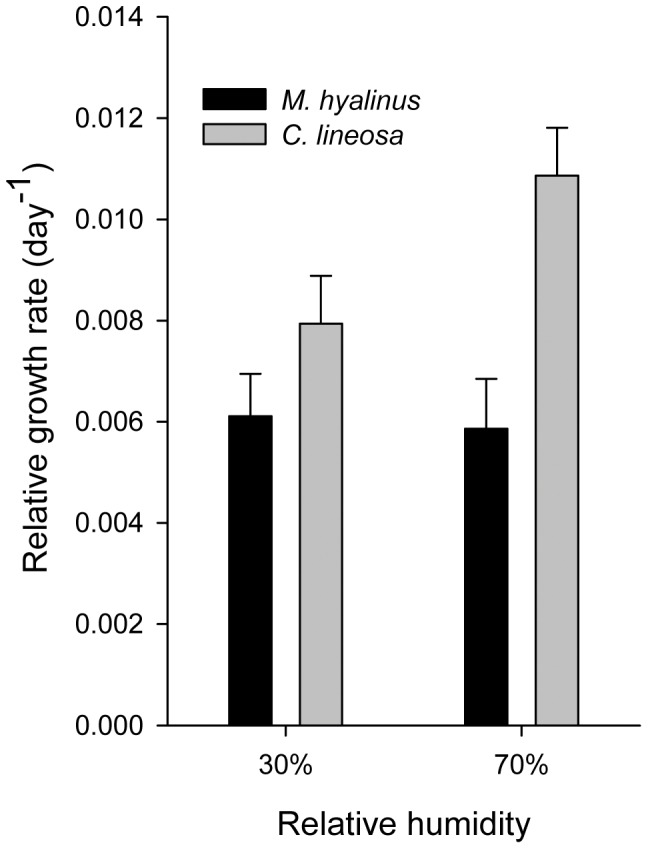
Relative growth rate of fed individuals. In the control group (which was fed regularly), *C. lineosa* gained mass faster than *M. hyalinus* (F_1, 42_ = 6.61, P = 0.013).

In the starved treatments (Fig. 7), *C. lineosa* lost body mass slower than *M. hyalinus* (F_1,67_ = 7.00, P = 0.01). Both species lost mass faster in the dry-air treatment (F_1,67_ = 22.462, P<0.001). In the control (fed) groups (Fig. 8), *C. lineosa* showed a faster relative growth rate than *M. hyalinus* (F_1,42_ = 6.61, P = 0.013).

## Discussion

Classical ecological studies discussing specialization usually focus on species’ performance along one niche axis, overlooking underlying mechanisms of species co-occurrence and coexistence [3]. This calls for a new effort to explore ecological specialization along more than one niche axis, combined with exposure to different types of environmental stress. As a model system to explore this new direction, we used two antlion species varying in their habitat utilization spectrum, a habitat generalist and a habitat specialist [33]. Notably, the foraging performance of the habitat generalist was found to be better than that of the habitat specialist in both sand and loess-derived soil types [33]. We aimed at testing whether the habitat specialist has an advantage over the habitat generalist along other niche axes, or if its poorer performance has been leading it towards an evolutionary dead-end. The three additional niche axes examined were high temperature, food abundance (feeding vs. starvation) and desiccation stress. Our results indicate niche separation along the temperature and food abundance axes, but not along the axis of relative humidity. Specifically, we illustrate that the habitat specialist, *C. lineosa*, has an advantage over the habitat generalist, *M. hyalinus*, at high temperatures, exhibiting a higher survival rate, increasing its pit size, and improving both its response time to prey and its prey capture success. From the perspective of thermal adaptation [57], our results suggest that *C. lineosa* is a high-temperature specialist, relative to the low-temperature generalist *M. hyalinus*. *C. lineosa* is also more efficient in its energy use, losing less body mass than *M. hyalinus* during prolonged starvation, and gaining mass more efficiently than *M. hyalinus* when fed regularly. Remarkably, this advantage of the habitat specialist over the habitat generalist is evident at high and low levels of desiccation stress. These results indicate that the previously reported apparent superiority of the habitat generalist [33] does not manifest itself along other niche axes; rather, the habitat specialist holds a set of traits that give it an advantage under harsh environmental conditions. All of the above suggests that microclimate (microhabitat) differences play an important role in maintaining the coexistence of these two species.

Antlions seemed to experience gradual heating as a harsher treatment than sudden heating, losing more mass in the former. In their natural environment, antlions experience large day/night temperature fluctuations, causing behavioural changes as a response [58]. It is possible that the gradual change in temperature imposed in our experiment was too moderate to evoke behavioural or physiological responses which could minimize energy expenditure and water loss. The fact that this differential effect of heating rate between treatments was evident only in the frequently disturbed cups may suggest that it can be discerned only when activity levels are high. Indeed, the frequent disturbance of antlions to enable weighing forced them to reconstruct their pits several times, maintaining high activity levels. Differences between the two species were still evident, as *M. hyalinus* suffered more severely from the heating in the warmer phase of the experiment, in terms of body mass loss. In the undisturbed cups, differences between the species were consistent with our other experiments, with *M. hyalinus* constructing larger pits, losing mass more rapidly, and decreasing its pit size over time, compared to the relatively stable pit size of *C. lineosa*. Since the gradual heating is experienced by the antlions as the harsher treatment (faster mass loss), response times also agree with other results: *C. lineosa*, which is better adapted to harsh conditions, responds faster in the gradual heating treatment, while *M. hyalinus* responds faster in the sudden heating treatment. These results reinforce the notion that *C. lineosa* is a high-temperature specialist, while *M. hyalinus* is a low-temperature generalist.

Classical studies examining local adaptation usually compared organisms or populations originating from different climates [27], [59], but this study brings forth the important effect of microclimate – *C. lineosa*, the species usually exposed to high temperatures, also performs better under these conditions, and thus *M. hyalinus* loses its advantage. These findings point towards a separation between the two species along the thermal niche axis. The effect of temperature on foraging activity has also been demonstrated in other insects, most commonly in ants [60], [61]. Arid conditions are characterized not only by high temperature, but also by lower relative humidity, leading to increased water loss. We did not find significant differences between the two species in the effect of relative humidity, as both species seemed to suffer similarly from the desiccation stress. *C. lineosa* did not seem to suffer less at low relative humidity, as might be expected, but perhaps exposure to higher temperatures (for a shorter time period), combined with the low relative humidity, may have brought forth different results. Perhaps in a short-term experiment with high temperatures and high relative humidity, *M. hyalinus* would have performed better than under dry conditions. It is important to note that the antlions’ exposure to high temperature in the lab is different than field conditions - under lab conditions the sand in each antlion’s cup heats up in a relatively uniform fashion, and the antlions can’t escape the high temperature by burrowing deeper into the soil, as is evident in field studies [58], [62]–[64]. Therefore, the exposure to high temperature is more extreme in the laboratory than in the field. For this same reason, we set the long-term survivable starvation temperature to 30°C, which is closer to the daily mean temperature in the field than to the daily maximum.

*C. lineosa* was also found to be more efficient than *M. hyalinus* – under constant food supply, *C. lineosa* grows faster. Possible explanations for this may include a lower basal metabolic rate or better growth efficiency (i.e., the efficiency of converting prey mass to predator mass). Under starvation, *C. lineosa* lost mass slower than *M. hyalinus*, a result that may indicate a decreased metabolic rate, different metabolic fuel utilization (e.g., [65]) or behavioural changes (i.e., decreased pit-building and maintenance activity). We interpret this separation along the food abundance axis to mean that *C. lineosa* may be able to better handle sporadic feeding, and exploit prey efficiently even when it is encountered intermittently, thus exhibiting a better ability to persist in a variable environment. This ability is specifically important in sit-and-wait predators, which suffer from fluctuations in prey abundance much more than actively searching predators [45].

Like other sit-and-wait predators, such as spiders, antlions are capable of dramatically reducing their metabolic rates [46], [47]. Since not all species and populations experience the same fluctuations in prey abundance [48], the response to starvation may be dependent on habitat-of-origin. Indeed, Arnett and Gotelli [28] have illustrated that antlion populations originating from temperate regions had better starvation endurance than populations originating from sub-tropical regions. In our experiment, *C. lineosa*, originating from a harsher microhabitat, seems to have a lower basal metabolic rate than *M. hyalinus*, irrespective of food abundance. Note that the conditions antlions experienced in our experiments were extreme, and in some cases even higher than seasonal means in the field. However, we still find our results to be ecologically relevant, since natural selection exerts its most dramatic influence at the edges of the organism’s tolerance limits, even if exposure to these edges happens only once every few generations [66], [67].

To conclude, this work exemplifies a comprehensive approach to examining local adaptation, bringing forth the importance of differences in microclimates in promoting species coexistence, examining several niche axes, and exposing model organisms to extreme experimental conditions, in order to examine the limits of each species’ niche. Future directions for this research should include an exploration of the physiological and molecular mechanisms underlying *C. lineosa*’s superior tolerance to high temperatures. These mechanisms could include cuticle lipid composition and permeability to water loss [68], [69], differences in metabolic rates and metabolic fuel utilization (e.g., [65]), and different expression levels of heat-related proteins, such as HSPs [70], [71].
